# Implementation of an Emergency Medical Service–Integrated Teleconsultation Follow-Up Unit for Home-Based Patients With Suspected COVID-19 During Epidemiological Uncertainty in an Island Territory: Retrospective Descriptive Study

**DOI:** 10.2196/88161

**Published:** 2026-08-05

**Authors:** Florian Negrello, Alexis Fremery, Melina Baala, Albert Brizio, Laurent Villain-Coquet, Benjamin Bouinoune, Rishika Banydeen, Papa Gueye

**Affiliations:** 1 Emergency Department, University Hospital of Martinique, CS 90632, Fort de France, 97200, Martinique, +596 596552015; 2 Emergency Department, University Hospital of French Guiana, Cayenne, French Guiana; 3 General Practice Department, French West Indies University, Fort de France, Martinique

**Keywords:** epidemics, COVID-19, emergency medical services, call centers, continuity of patient care, emerging infectious disease

## Abstract

**Background:**

Epidemiological and biological risks frequently expose Caribbean territories to emerging infectious threats. Martinique, a French overseas territory, is particularly vulnerable due to its tropical climate, insular geography, and recurrent exposure to arboviral epidemics. During exceptional health crises such as the COVID-19 pandemic, health care systems must rapidly adapt to a potentially sustained patient influx, evolving scientific knowledge, and heightened population anxiety. In March 2020, following the first confirmed COVID-19 cases in Martinique, the emergency medical service (EMS) implemented a teleconsultation follow-up unit dedicated to home-based patients with suspected SARS-CoV-2 infection, in the context of uncertainty regarding disease progression.

**Objective:**

This study aimed to evaluate the health and psychological impact of this EMS-based teleconsultation follow-up unit during the first COVID-19 wave, in order to assess its potential as an organizational response strategy for future infectious health emergencies.

**Methods:**

We conducted a single-center, retrospective, descriptive study including all adult patients monitored by the EMS COVID-19 teleconsultation unit during the first wave of the COVID-19 pandemic, between March 10 and May 31, 2020. Patients were initially triaged through the EMS call center and followed remotely using a standardized daily questionnaire. Follow-up frequency was determined according to clinical presentation. Collected data included sociodemographic characteristics, medical history, symptoms, polymerase chain reaction (PCR) testing status, clinical outcomes (recovery, hospitalization, or death), anxiety levels assessed using a 5-point Likert scale at the beginning and end of follow-up, and satisfaction measured on a 10-point numeric scale.

**Results:**

Among 1255 patients monitored during the study period, 908 met inclusion criteria (mean age 45, SD 17 years; 57.6% female). Most patients (n=724, 79.7%) had no prior medical history. The most frequently reported symptoms were fever (n=177, 19.5%), respiratory difficulties (n=165, 18.2%), cough (n=152, 16.7%), myalgia (n=114, 12.6%), and diarrhea (n=91, 10.0%). Only 10.6% of patients underwent PCR testing due to limited availability during the early epidemic phase, of whom 62.5% tested positive. During follow-up, 68 patients (7.5%) required hospitalization, and 2 (0.2%) died during their hospital stay. The presence of at least 1 pre-existing medical condition and older age were both significantly associated with hospitalization for suspected COVID-19 (*P*<.001). Among 590 respondents, mean anxiety scores decreased significantly from 3.9 (SD 0.8) at baseline to 1.4 (SD 1.1) at the end of follow-up, representing a 64.1% reduction (*P*<.001). Overall patient satisfaction with the teleconsultation service was high (mean score 8.7, SD 1.2, of 10).

**Conclusions:**

In the context of a novel epidemic with limited diagnostic and therapeutic knowledge, an EMS-integrated teleconsultation follow-up system enabled safe outpatient management while significantly reducing patient anxiety and preserving hospital resources. This approach may represent a scalable organizational model for maintaining access to care and supporting population reassurance during future infectious disease emergencies in geographically constrained or resource-limited settings such as small island territories.

## Introduction

Epidemiological and biological risks refer to situations with a high probability of leading to major health crises, and the development of “one health” strategies represents a key preventive approach, particularly against emerging or re-emerging zoonoses [1,2]. The Caribbean is considered a high-risk area because of its tropical climate, its proximity to the primary forests and wildlife of South America, its role as a major exchange hub in the region, and its insular isolation [3]. Martinique, a French overseas territory in the Lesser Antilles particularly affected by arboviral diseases, has had to regularly adapt its health care system to recurrent and emerging epidemics [3,4]. Since the 2000s, it has witnessed significant and frequent dengue epidemics (2001, 2005, 2007, 2010, 2013, and 2020), as well as new arboviral diseases such as chikungunya in 2014 and Zika in 2016. In addition, frequent health alerts related to transmissible infectious diseases have required public health responses, including severe acute respiratory syndrome in 2003, H1N1 influenza in 2009, Ebola in 2014, SARS-CoV-2 in 2019, mpox (monkeypox) in 2022, and Oropouche virus in 2024 [5].

Specific characteristics of such epidemics in exceptional health situations include a distinctive pattern of patient influx characterized by exponential transmission over an extended period [6], the need for infectious disease research to identify novel pathogens and implement collective protection measures aimed at limiting the number of cases [7], a possible weakening of the health care system due to health care workers falling ill and experiencing stress [8,9], and, finally, clear communication to ensure that the population maintains adaptive behaviors and an acceptable level of anxiety, particularly when the infectious agent is unknown [10,11]. The most recent major epidemic in the French West Indies exhibiting these characteristics was the COVID-19 pandemic. This epidemic triggered a global social and health crisis, profoundly disrupting health care systems [12]. Caribbean territories were particularly affected, initially marked by distrust of the health care system and significant anxiety, followed by a surge in cases and high mortality [13].

In response to the first cases of COVID-19 in Martinique in March 2020, adapting the organization of the health care system became essential. The emergency medical service (EMS) call center in Martinique established a teleconsultation platform within the first week after the first diagnosed case to monitor outpatients with suspected SARS-CoV-2 infection. This initiative aimed to prevent emergency services and hospital departments from being overwhelmed by nonessential consultations, while ensuring continuous remote medical monitoring to address patient needs and concerns regarding this novel and poorly understood epidemic and minimizing the risk of viral transmission. The follow-up unit was located within the EMS at the University Hospital of Martinique and was supervised by an EMS physician. It operated daily during daytime hours, from March 10 to May 31, 2020, covering the first COVID-19 wave. The unit was staffed by 4 residents and 2 to 3 physicians from the hospital’s outpatient consultation services, whose regular activities had been temporarily suspended. A dedicated app was developed, incorporating a standardized daily questionnaire used by all staff for individual follow-up. During daily staff meetings, infectious disease specialists reviewed patients whose conditions had worsened and arranged hospital admission when necessary.

The objective of this study was to evaluate the health and psychological impact of this teleconsultation follow-up unit for home-based patients during a period of epidemiological uncertainty, with the aim of formalizing it as a response strategy for exceptional infectious health crises, particularly when knowledge of the disease remains limited.

## Methods

### Study Design

This was a single-center, retrospective, descriptive study conducted at the EMS of the University Hospital of Martinique during the first wave of the COVID-19 pandemic, between March 10 and May 31, 2020.

The primary objective was to describe the evolving profile of patients managed by the EMS follow-up unit. All patients over 18 years of age monitored by the COVID-19 follow-up unit of EMS of Martinique were included after providing consent at the start of follow-up. Patients who failed to respond to follow-up calls for more than 3 consecutive days were subsequently excluded.

Data were collected from the EMS software (Centaure 15; Nexpublica Switzerland AG), which compiles medical regulation records, and from the COVID-19 patient follow-up application database, which contains all follow-up teleconsultations. All patients included had initially contacted EMS and were triaged by emergency medical operators before being transferred to the follow-up unit. They were then contacted according to predefined protocols:

- Asymptomatic contact cases: contacted every 3 days for up to 7 days (day 1, day 4, and day 71)
- Symptomatic cases with flu-like symptoms and without respiratory symptoms: monitored daily until symptoms resolved for three consecutive days
- Symptomatic cases with dyspnea: referred to infectious disease specialist staff for evaluation and, if necessary, transfer to the emergency department for clinical evaluation

Patient follow-ups were conducted using a standardized questionnaire within the monitoring app. Collected data included sex, age, city of residence, medical history, polymerase chain reaction (PCR) tests performed, symptoms, follow-up outcome (recovery, hospitalization, or death), anxiety level at the beginning and end of follow-up, and satisfaction at the end of follow-up.

Anxiety level, added later in the process, was assessed using a numeric rating scale based on a Likert scale from 0 (not anxious) to 5 (extremely anxious). Satisfaction level was measured on a numeric scale from 0 (very dissatisfied) to 10 (very satisfied).

### Analysis

Continuous variables were described using means and SDs, while categorical variables were summarized using frequencies and percentages. *χ*² tests or Student *t* tests were used to evaluate associations between patients’ medical history and hospitalization status, as well as to assess reductions in anxiety scores before and after the intervention. A significance level of .05 was used.

### Ethical Considerations

This study was conducted in accordance with the Declaration of Helsinki and was approved by the Institutional Review Board of the University Hospital of Martinique on June 3, 2021 (No. 2021/108), as well as by the French National Commission on Informatics and Liberty. Consent to participate was obtained at the beginning of the follow-up. All data were anonymized prior to analysis and handled in accordance with the General Data Protection Regulation. Participants did not receive any financial compensation for their participation in the study.

## Results

**Figure 1.**
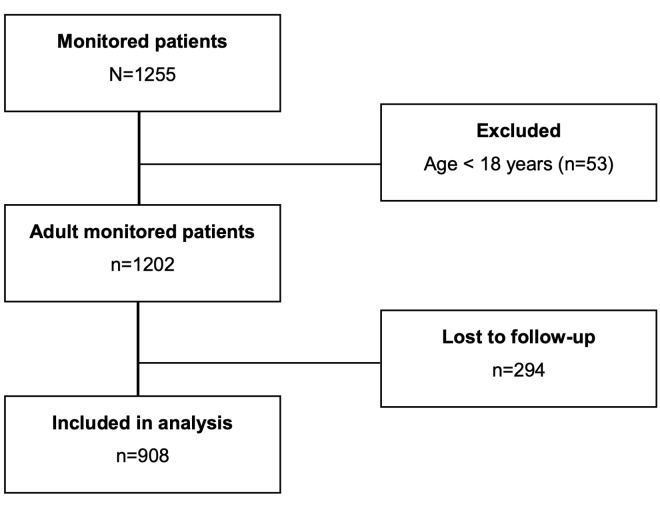
Flowchart of participant selection for analysis.

The COVID-19 follow-up unit of the EMS managed 1255 patients between March 10 and May 31, 2020. After applying noninclusion and exclusion criteria, 908 patients were included (Figure 1).

**Figure 2.**
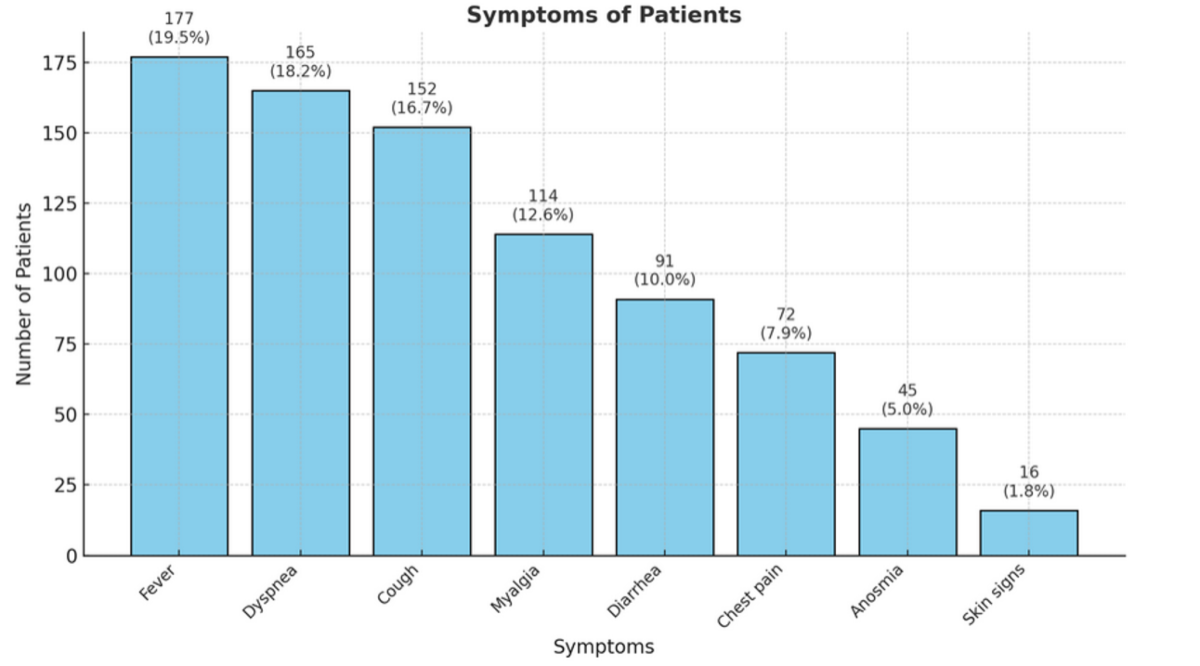
Symptoms of patients with suspected COVID-19 (N=908) monitored by the emergency medical services (EMS) follow-up unit, University Hospital of Martinique (March 10 to May 31, 2020). The remaining patients reported no symptoms or had no symptoms recorded.

The characteristics of the study population (N=908) are presented in Table 1. The population was predominantly female (n=523, 57.6%), with a mean age of 45 (SD 17) years. Among the monitored patients, 724 (79.7%) reported no prior medical history. The most frequent symptoms were fever (n=177, 19.5%), respiratory difficulties (n=165, 18.2%), cough (n=152, 16.7%), myalgia (n=114, 12.6%), and gastrointestinal symptoms such as diarrhea (n=91, 10.0%) (Figure 2). Only 96 patients (10.6%) underwent COVID-19 PCR testing due to limited availability at the onset of the epidemic. Of these, 60 tests were positive, representing 62.5% of those performed. Of the 908 included patients, 68 (7.5%) were hospitalized during follow-up, and 2 patients (0.2%) died, both during hospitalization. The presence of at least 1 pre-existing medical condition and older age were each significantly associated with hospitalization for suspected COVID-19 (Table 2).

Anxiety related to the emerging COVID-19 pandemic was a notable concern during follow-up. A significant reduction in patient anxiety was observed after monitoring by the follow-up unit at the University Hospital of Martinique (590 patients surveyed). Before follow-up, the mean anxiety level was 3.9 (SD 0.8) on a 5-point scale. After follow-up, this level decreased to 1.4 (SD 1.1), representing a 64.1% reduction in perceived anxiety (*P*<.001). Finally, the COVID-19 telephone monitoring unit of the Service d’Aide Médicale Urgente at the University Hospital of Martinique received a mean patient satisfaction score of 8.7 (SD 1.2) on a 10-point scale.

**Table 1.** Characteristics of patients with suspected COVID-19 (N=908) monitored by the emergency medical services (EMS) follow-up unit, University Hospital of Martinique (March 10 to May 31, 2020).

Characteristics	Participants, n (%)
Sex	
Female	523 (57.6)
Male	385 (42.4)
Age (years)	
18‐30	180 (19.8)
30‐40	199 (21.9)
40‐50	161 (17.7)
50‐60	187 (20.6)
60‐70	94 (10.4)
70‐80	53 (5.8)
80‐90	26 (2.9)
>90	8 (0.9)
Living area	
Home	896 (98.7)
Medico-social institution	12 (1.3)
Medical history^a^	
Chronic respiratory disease	67 (7.4)
Asthma	56 (6.2)
Chronic kidney failure	3 (0.3)
Diabetes	32 (3.5)
Cancer	8 (0.9)
Sickle cell disease	1 (0.1)
Obesity	29 (3.2)
Bedridden	33 (3.6)
No medical history	724 (79.7)

a Percentages may exceed 100% as patients could report multiple comorbidities.

**Table 2.** Medical history of patients with suspected COVID-19 (N=908) monitored by the emergency medical services (EMS) follow-up unit of the University Hospital of Martinique, stratified by follow-up outcome (March 10 to May 31, 2020).

Variable	Nonhospitalized (n=840)	Hospitalized (n=68)	*P* value
Age (years), mean (SD)	44.5 (17)	57.2 (17)	<.001
No medical history, n (%)	692 (82.4)	32 (47.1)	<.001
Chronic respiratory disease, n (%)	61 (7.3)	6 (8.8)	.66
Asthma, n (%)	52 (6.2)	4 (5.9)	>.99
Chronic kidney disease, n (%)	1 (0.1)	2 (2.9)	.02
Diabetes, n (%)	24 (2.9)	8 (11.8)	.002
Cancer, n (%)	4 (0.4)	4 (5.9)	.002
Obesity, n (%)	26 (3.1)	3 (4.4)	.46
Bedridden, n (%)	22 (2.6)	11 (16.2)	<.001
Living in long-term care facility, n (%)	10 (1.2)	2 (2.9)	.22

## Discussion

### Principal Findings

Emerging epidemics pose major challenges to health care systems, particularly during periods of health uncertainty [14]. Teleconsultation offers an innovative and effective solution to maintain access to care while reducing the risk of infection [15]. The EMS follow-up teleconsultation unit was urgently established in the context of uncertainty regarding COVID-19 and its evolution. During the first epidemic wave in Martinique, knowledge about the disease was limited, prevention and diagnostic tools were underdeveloped, and public anxiety was high.

The urgency of establishing this system led to the implementation of a straightforward telephone follow-up approach. While videoconferencing is generally recommended for teleconsultations [16], the immediacy of the COVID-19 pandemic’s onset did not allow for the inclusion of a video component. Moreover, a multimedia teleconsultation strategy with integration of vital sign reporting through tracking apps might have provided higher-quality care but could have posed a substantial barrier to adoption, particularly among older or socially vulnerable populations in Martinique [17-20]. Nevertheless, the safety of this system was ensured by an experienced team trained in telephone-based tele-evaluation.

### Teleconsultation During Emerging Epidemics

In the context of an emerging epidemic, rapid spread of infection is a major risk. Teleconsultation can help reduce patient movements to high-risk environments such as emergency departments [21]. In our case, it also addressed a new societal factor: the government-mandated lockdown, which complicated transport due to the suspension of public transportation. Teleconsultation additionally ensured regular medical follow-up, particularly for vulnerable patients in isolated areas [22]. Epidemics can disrupt health care infrastructure, leading to shortages of supplies, sudden increases in workload, or the isolation of specific regions. The risk of transmission to health care workers also remains significant, and teleconsultation can help protect this crucial workforce in epidemic contexts.

During uncertain epidemics such as COVID-19 in its early stages, it is essential to rapidly identify suspected cases and provide appropriate recommendations. Teleconsultation serves as an efficient tool for initial remote triage [23]. Physicians can assess symptoms, direct patients to appropriate centers for diagnostic testing or advanced care, and provide remote advice. This early triage helps prevent health care facilities from being overwhelmed and reduces the risk of contamination in clinical settings, thereby preserving the quality of care and the management of severe cases. Teleconsultation allows nonurgent or concerned patients to receive assessments remotely, preserving hospital capacity for critical cases and reducing system strain. In our cohort, most patients completed follow-up at home, whereas hospitalized patients were older and had more comorbidities, consistent with findings from prior studies [24,25].

During rapidly evolving epidemics such as COVID-19, teleconsultation enables agile adaptation of treatments and remote monitoring. Physicians can track symptom progression, adjust prescriptions according to updated medical guidelines, and collect essential patient information. This approach is especially valuable in the early stages of an epidemic, as treatment protocols can evolve quickly with new evidence and clinical experience. A major initial challenge was diagnosis, which relied primarily on clinical assessment due to the limited availability and long turnaround times of PCR testing. Clinical variability, compounded by high public anxiety, justified the follow-up system implemented by the EMS. Anxiety was a major concern during this health crisis [26,27]. Telephone follow-up revealed a significant reduction in patient anxiety, suggesting that teleconsultation services can play a role not only in medical care but also in psychological support during epidemics. Despite the potential for satisfaction bias, the high level of reported patient satisfaction supports the idea that this type of service can benefit emotionally vulnerable populations during health crises.

### Limitations

This study has several limitations. First, its retrospective, single-center design limits the generalizability of the findings, as the teleconsultation system was implemented within the specific geographic and health care context of Martinique. Second, diagnostic confirmation was limited because PCR testing was not widely available during the early phase of the epidemic, and many patients were managed based on suspected infection; nevertheless, similar constraints may be encountered during the emergence of a new infectious disease or during large-scale epidemics. Third, some data, including anxiety levels and satisfaction, were self-reported during telephone calls with a numeric scale and did not use a validated psychological measure, and they may be subject to reporting bias.

### Conclusions

In the context of a novel and uncertain epidemic, teleconsultation, as implemented during the COVID-19 pandemic by the EMS at the University Hospital of Martinique, appears to be a valuable tool for addressing public health needs while minimizing transmission risks. It helps ensure continuity of care, optimize patient flow, and provide close individual monitoring, thereby alleviating pressure on health care systems. Although this organization was implemented within a specific geographic, organizational, and health care context, some components, such as the integration of teleconsultation within EMS regulation and the use of remote monitoring to manage selected patients at home, may represent adaptable approaches that could inform preparedness and response strategies during future epidemiological or biological health crises.

## Abbreviations

EMS: emergency medical service
PCR: polymerase chain reaction
